# CTX-M-9 group ESBL-producing *Raoultella planticola* nosocomial infection: first report from sub-Saharan Africa

**DOI:** 10.1186/s12941-020-00380-0

**Published:** 2020-08-17

**Authors:** Tafese Beyene Tufa, Andre Fuchs, Torsten Feldt, Desalegn Tadesse Galata, Colin R. Mackenzie, Klaus Pfeffer, Dieter Häussinger

**Affiliations:** 1 Asella Teaching and Referral Hospital, College of Health Sciences, Arsi University, P.O. Box 04, Asella, Ethiopia; 2 Hirsch Institute of Tropical Medicine, P.O. Box 04, Asella, Ethiopia; 3 Department of Gastroenterology, Hepatology and Infectious Diseases, Düsseldorf University Hospital Center, Moorenstr. 5, 40225 Düsseldorf, Germany; 4 Institute of Medical Microbiology and Hospital Hygiene, Düsseldorf University Hospital Centre, Universitätsstr. 1, 40225 Düsseldorf, Germany

**Keywords:** *Raoultella planticola*, Nosocomial infection, Antimicrobial resistance, Extended spectrum β-lactamases, ESBL, CTX-M-9 group, Africa, Ethiopia

## Abstract

**Background:**

*Raoultella* are Gram-negative rod-shaped aerobic bacteria which grow in water and soil. They mostly cause nosocomial infections associated with surgical procedures. This case study is the first report of a *Raoultella* infection in Africa.

Case presentation

We report a case of a surgical site infection (SSI) caused by *Raoultella planticola* which developed after caesarean section (CS) and surgery for secondary small bowel obstruction. The patient became febrile with neutrophilia (19,157/µL) 4 days after laparotomy and started to develop clinical signs of a SSI on the 8^th^ day after laparotomy. The patient continued to be febrile and became critically ill despite empirical treatment with ceftriaxone and vancomycin. *Raoultella* species with extended antimicrobial resistance (AMR) carrying the CTX-M-9 β-lactamase was isolated from the wound discharge. Considering the antimicrobial susceptibility test, ceftriaxone was replaced by ceftazidime. The patient recovered and could be discharged on day 29 after CS.

**Conclusions:**

*Raoultella planticola* was isolated from an infected surgical site after repeated abdominal surgery. Due to the infection the patient’s stay in the hospital was prolonged for a total of 4 weeks. It is noted that patients undergoing surgical and prolonged inpatient treatment are at risk for infections caused by *Raoultella*. The development of a SSI caused by *Raoultella planticola* with extended AMR has to be assumed to be a consequence of ineffective antibiotic utilization. The presented case advices that rare bacteria as *Raoultella* should be considered as potential cause of nosocomial SSI with challenging treatment due to high levels of AMR.

## Background

*Raoultella* are Gram-negative rod-shaped aerobic bacteria growing in water and soil. They can also be detected in the human gastrointestinal tract (GIT) or upper respiratory tract (URT) and are a rare cause of mostly nosocomial infections in humans. They were defined as a new genus in the family of *Enterobacteriaceae* in 2001, based on gene sequences of its 16S rRNA and *rpoB* gene [1]. *Raoultella *can grow at wide range of temperature (4 °C to 44.5 °C) and do not produce gas from lactose at 44.5 °C. All *Raoultella* isolates are resistant to ampicillin due to the over expression of chromosomally encoded class-A β-lactamase [2].

*Raoultella planticola, R. ornithinolytica*, *R. terrigena*, and *R. electrica* are medically relevant *Raoultella* species [3–6], with *R. planticola and R. ornithinolytica* currently being most commonly reported from clinical samples. Factors contributing to the pathogenesis of diseases caused by the genus of *Raoultella* share similarities with those of *Klebsiella* and include lipopolysaccharides, polysaccharide capsules, fimbriae, siderophores [7], toxins [8], hydrolytic enzymes, and bacteriocins [9]. *Raoultella* species are also able to form biofilms [10]. In contrast to *Klebsiella* species, *Raoultella* species harbour histidine decarboxylase, enabling the bacteria to produce histamine. This information might be used for species differentiation [11, 12].

Following phenotypic and biochemical microbiological methods only, *Raoultella* species are most likely being underreported due to the difficult differentiation from *Klebsiella* species. Over recent years, the identification rate has improved by increased utilization of matrix-assisted laser desorption/ionization-time of flight mass spectrometry (MALDI-TOF MS) [13].

*Raoultella planticola* is not well known as human pathogen. Literature search revealed 87 reported cases of *R. planticola*-related infections. Here, bloodstream infections (32 cases), urinary tract infection (16 cases), and pneumonia (11 cases) are most frequent. Among abdominal foci of *Raoultella*-infections, 5 cases of cholangitis, 3 cases of pancreatitis, 3 cases of cholecystitis, 3 cases of surgical site infection (SSI), 2 cases of secondary bacterial peritonitis, and a single case of enterocolitis have been described. Antimicrobial resistance (AMR) of *Raoultella* causing human infections has not been analysed systematically. However, *Raoultella* species harbouring extended spectrum β-lactamase (ESBL) and carbapenemase genes have been reported [14–17]. Most of the cases were reported in Europe and the USA. To our best knowledge, this report is the first description of an SSI caused by *R. planticola* with multidrug resistance (MDR) in Africa.

## Case presentation

### Initial presentation

A 17-year-old previously healthy pregnant woman presented to Asella Teaching and Referral Hospital (ATRH) delivery ward in Asella, Central Ethiopia. Upon admission she appeared healthy, without any signs of infection or life-threatening disease. Caesarean section (CS) was indicated due to posterior cephalic position of the child and large fetal size. Lower uterine transverse CS was performed and a healthy male neonate delivered.

### Development of surgical site infection

For the first three days after delivery, the patient recovered well from her surgery. However, on the 4^th^ day she developed cramping abdominal pain, constipation with clinical signs of ileus and an elevated body temperature (T) of 37.8 °C. After physical examination and abdominal X-ray revealed signs of small bowel obstruction, emergency laparotomy was performed. Intra-operative findings were a purulent peritonitis due to a volvulus of the cecum with formation of a gangrene. Peritoneal drainage and lavage and a right hemicolectomy with primary ileo-transverse anastomosis were performed. The postoperative course for the first days was uneventful.

On her 5th day after laparotomy (and 9th after CS) the patient developed shortness of breath with mild hypotension, tachycardia, tachypnea and fever (blood pressure (BP) 110/70 mmHg, pulse rate (PR) 108/min, RR 40/min, T 38.5 °C). Breath sounds were clear with good bilateral air entry. Abdominal examination revealed passage of faecal matter from the surgical site. Complete blood count (CBC) showed leucocytosis (21.5 × 10^3^/µl) with an increased fraction of neutrophils (89.1%). Platelet count and haemoglobin level where within normal range. There was no growth in a blood culture for a total incubation period of 5 days (1 bottle, local production).

### Further course and treatment

The patient was diagnosed with a suspected intestinal anastomotic leak and empiric parenteral antibiotic treatment was started according to local guidelines with 1 g ceftriaxone plus 1 g vancomycin daily. Re-laparotomy on the same day revealed intraperitoneal pus and faeces due to an anastomotic dehiscence with perforation of the distal ileum about 50 cm from the previous anastomosis. After dissection of the insufficient anastomosis, resection of necrotic intestine and peritoneal lavage, re-anastomosis and closure of the abdominal cavity were performed.

**Fig. 1:**
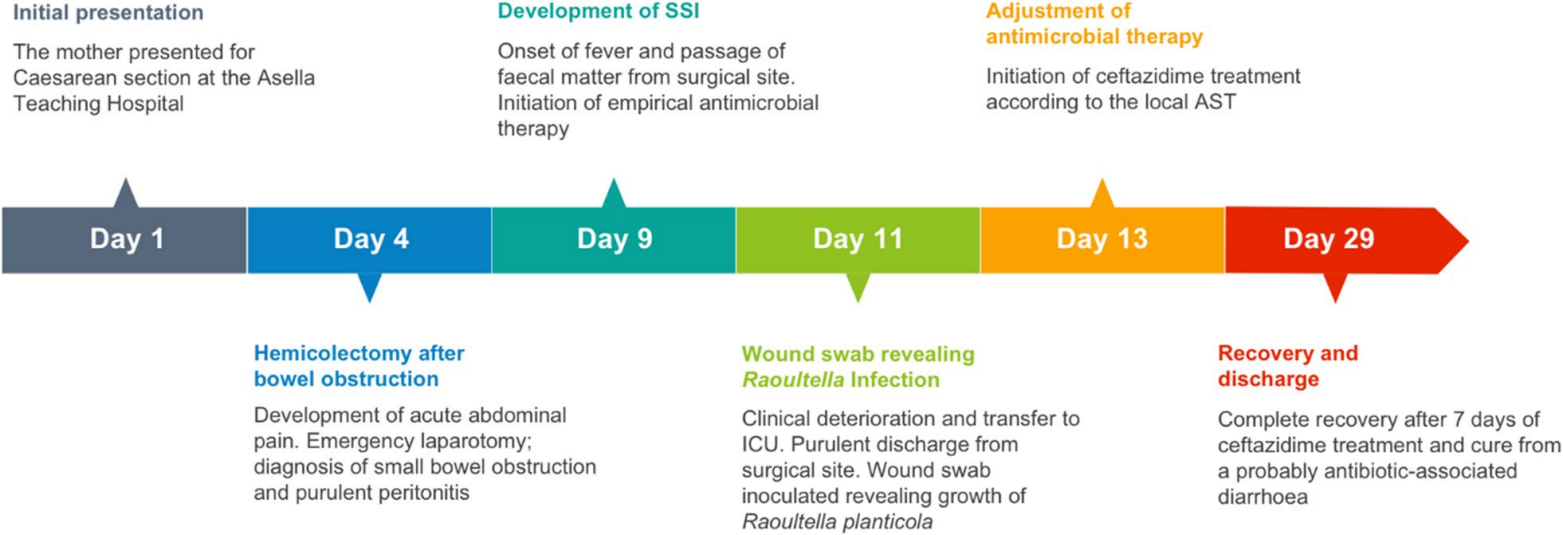
Timeline of the development and course of the surgical site infection

On the 6^th^ day post re-laparotomy (11 days post first laparotomy and 15 days after CS), the patient was transferred to intensive care unit and she developed purulent discharge from the surgical site. At this time, a wound swab was taken for microbiological diagnostics. The culture revealed growth of *Raoultella* species and the previous antibiotic treatment was adjusted according to the drug susceptibility test result (see Table 1) by replacement of ceftriaxone with ceftazidime. After 7 days of parenteral antibiotic treatment with this new regimen (ceftazidime 1 g three times daily and vancomycin 1 g once daily) the patient developed frequent watery diarrhoea and bilateral lower extremity swelling. Because of suspected *Clostridium difficile* enteritis (diagnostic tests for *Clostridium difficile* are not available), intravenous antibiotics were discontinued and the patient was started on oral metronidazole. Along with easing of the diarrhoea, the patient recovered and could be discharged in good condition on the 29^th^ day after CS. In general the case was summarized by (Fig. 1).

**Table 1:** Results of antimicrobial susceptibility testing of *Raoultella planticola* isolated strain

Name of antimicrobial substance	Kirby–Bauer disc diffusion test^a^		VITEK® 2 result	
	Diameter (mm)	EUCAST interpretation	MIC	EUCAST interpretation
Piperacillin	0	R	≥ 128	R
Piperacillin/tazobactam	21	S	8	S
Cefotaxime	0	R	8	R
Ceftazidime	22	S	≤ 1	S
Cefepime	22	I	≤ 1	S
Aztreonam	Not tested		2	I
Imipenem	26	S	0.5	S
Meropenem	27	S	≤ 0.25	S
Amikacin	20	S	≤ 2	S
Gentamicin	9	R	≥ 16	R
Tobramycin	12	R	8	R
Moxifloxacin	Not tested		2	R
Tigecycline	Not tested		1	S
Ciprofloxacin	17	R	1	R
Fosfomycin	Not tested		≤ 16	S
Colistin	Not tested		≤ 0.5	S
Trimethoprim/sulfamethoxazole	Not tested		≥ 320	R

*MIC* minimum inhibitory concentration; *R* resistant, *S* sensitive, *I* intermediate

^a^Results of antimicrobial susceptibility testing (AST) was done by using disc diffusion method at Asella, Ethiopia whereas VITEK was performed at institute of Medical Microbiology and Hospital Hygiene, Düsseldorf, Germany. Both results were interpreted by using European Committee on Antimicrobial Susceptibility Testing (EUCAST) version: 08.01

### Microbiology results

During the patient’s stay in the hospital, one blood culture and one wound swab from the surgical site were sent for microbiological culture. Despite the intraoperative finding of purulent peritonitis upon first laparotomy, no intraoperative swabs were ordered. The blood culture remained sterile after an incubation period of 5 days. The swab taken from the SSI 15 days after CS and before the 2^nd^ laparotomy was positive for Gram-negative rod-shaped bacteria. According to biochemical identification tests performed on site in Ethiopia, the isolated bacteria were identified as *Klebsiella oxytoca* (oxidase and methyl red negative; lactose, urease, citrate and indole positive). The isolate was exported to the Institute of Medical Microbiology and Hospital Hygiene at Heinrich Heine University Düsseldorf, Germany for confirmation, further identification and antimicrobial susceptibility testing (AST)”. Using MALDI-TOF (VITEK®-MS, bioMérieux, Marcy-l’Étoile, France), the bacteria was re-classified as *Raoultella planticola* with a likelihood of 99.9%.

The AST was done using Kirby–Bauer disc diffusion and VITEK methods. The results of the disc diffusion test and the VITEK® 2 (bioMérieux) investigation are described in (Table 1). For molecular resistance gene detection, polymerase chain reactions with primers described in (Table 2) were performed [18].

**Table 2:** Oligonucleotide sequences of the primer pairs for molecular resistant genes detection

Primer	Sequence (5ʹ–3ʹ)	Amplicon size (bp)
bla_SHV_ (F)	AGCCGCTTGAGCAAATTAAAC	786
bla_SHV_ (R)	GTTGCCAGTGCTCGATCAGC	
bla_TEM_ (F)	CATTTCCGTGTCGCCCTTATTC	846
bla_TEM_ (R)	CCAATGCTTAATCAGTGAGGC	
bla_CTX-M-1_ (F)	CGTCACGCTGTTGTTAGGAA	781
bla_CTX-M-1_ (R)	ACGGCTTTCTGCCTTAGGTT	
bla_CTX-M-2_ (F)	CTCAGAGCATTCGCCGCTCA	843
bla_CTX-M-2_ (R)	CCGCCGCAGCCAGAATATCC	
bla_CTX-M-9_ (F)	GCGCATGGTGACAAAGAGAGTGCAA	876
bla_CTX-M-9_ (R)	GTTACAGCCCTTCGGCGATGATTC	

Possible extended spectrum β-lactamases (ESBLs) coding genes were screened by using conventional polymerase chain reaction (PCR)

*F* forward, *R* reverse, *bp* base pairs

**Fig. 2:**
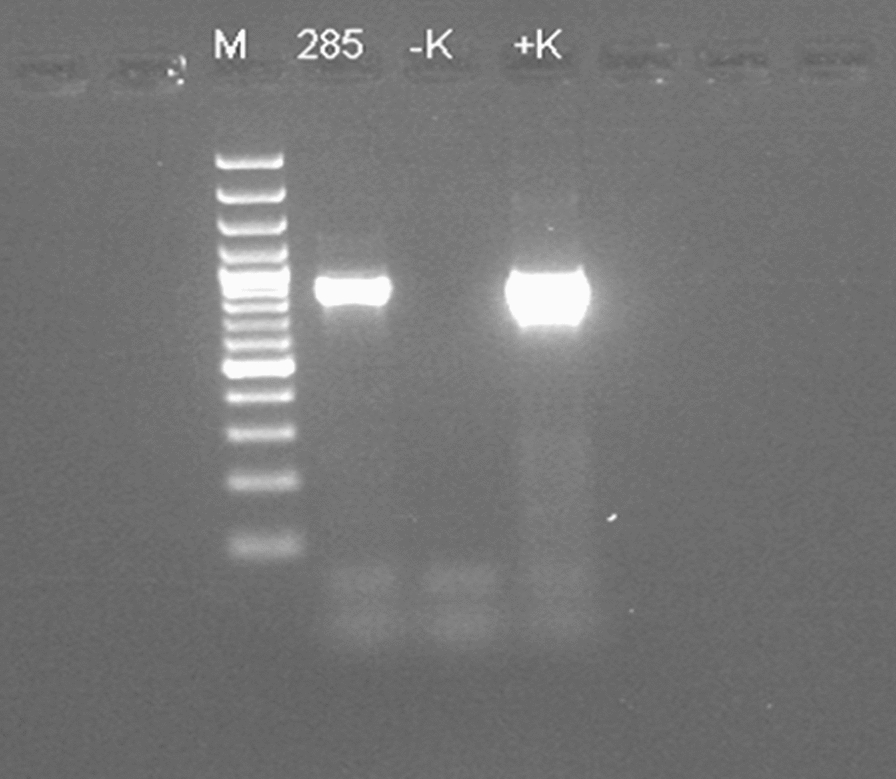
Picture of *bla*CTX-M-9 ESBL positive result. *bla*CTX-M-9 ESBL gene detected in the isolated *R. planticola* strain (with M = DNA ladder; −K = negative control; + K = positive control; 285 = code given for patient’s sample

CTX-M-9 group and TEM ESBL coding genes were detected and the isolated strain was identified as MDR (Fig. 2). However, ESBLs from the groups CTX-M-1, CTX-M-2 or the SHV β-lactamase were not detected.

## Discussion

Cases of infections caused by *Raoultella planticola* including infections with abdominal foci have been reported from different countries [19]. In addition, the isolation of *Raoultella* species as the causative agent of human infections containing ESBL and also carbapenemase genes have previously been reported. It is assumed that, to date, *Raoultella* species are often misdiagnosed as *Klebsiella* species in the context of restricted diagnostic capacities in many African laboratories. Therefore, and to our best knowledge, the described hospital-acquired SSI caused by an ESBL-producing strain of *R. planticola* is the first such case reported in Africa. The bacterial strain was isolated from a SSI after CS complicated by cecal volvulus with secondary peritonitis due to a breakdown of the primary anastomosis. Due to secondary infection the patient’s overall stay in the hospital was prolonged. It is noted that being immunocompromised, surgical procedures, long-term antibiotic therapy and prolonged stays in hospital have been described as risk factors to develop *Raoultella* infections [17, 20]. In our case report, the patient’s gastrointestinal tract has probably been colonized with *R. planticola* and the leakage of intestinal luminal contents and gut flora after anastomosis insufficiency was the likely route of the infection. It appears likely that, selection of resistant bacteria by the previous empirical antibiotic therapy with ceftriaxone, which was ineffective against identified *R. planticola*.

Cases of *Raoultella* infections can occur in many organ systems (e.g. urinary tract, gastrointestinal tract, respiratory tract) or at surgical sites. Bacteraemia, osteomyelitis, meningitis, cerebral abscess, mediastinitis, pericarditis, conjunctivitis, mandibular osteomyelitis and otitis caused by *Raoultella* have also been reported [20, 21]. In general, the most common microorganisms isolated from SSIs after small bowel surgery are aerobic Gram-negative enteric bacteria [14].

Regarding the management of intra-abdominal infections, therapy should focus on adequate source control and appropriate adjustment of antimicrobial therapy to individual patient factors. Empiric antimicrobial therapy is essential [22], however inappropriate antibiotic therapy may result in unfavourable outcome and in the selection of bacterial AMR bacteria. As seen in the described case, health care-associated infections caused by multi-resistant bacteria have to be considered which lead to the necessity of complex multidrug regimens [23]. For the selection of empirical antibiotic treatment, a length of hospital stay of 5 days has moderate specificity and very high sensitivity for predicting the presence of MDR bacteria [24].

Due to the high likelihood of infections caused by resistant bacteria, guidelines for empirical treatment of SSIs recommend utilization of broad spectrum antimicrobials (e.g. carbapenems) [25] which are frequently not available in resource-limited settings like Ethiopia. In the absence of carbapenems, piperacillin/tazobactam could be an option for the empiric treatment of high-risk intra-abdominal infections [26, 27], which lead to high rates of mortality [28].

The initial empirical treatment in the described case was a combination of vancomycin and ceftriaxone. It was shown to be ineffective according to the subsequently available AST results (see Table 1). The patient only recovered after the antibiotic treatment was adapted according to the AST, despite the local miss-identification of the causing pathogen as *Klebsiella* species. If AST results are not or not yet available, the initiation of antimicrobial treatment with substances active against most *Enterobacteriaceae* should be considered for critically-ill patients with secondary intra-abdominal infections [29]. In general, regional and local susceptibility profiles of common bacterial isolates should be available and considered before initiation of empiric antibiotic treatments. This becomes increasingly important because of rising resistance of Gram-negative bacteria circulating in the communities. Local guidelines considering common regional AMR patterns should be implemented and updated for the management of SSI and intra-abdominal infections.

From both clinical and natural environment, MDR strains of *R. planticola* and *R. ornithinolytica* were reported [30, 31]. In recent years, infections with *Raoultella* strains producing ESBL from the families TEM, SHV, and CTX-M have been described [32]. The ability to produce ESBL was also detected in *Raoultella* strains isolated from the hospital environment. From different clinical samples, AmpC β-lactamase-producing *R. ornithinolytica* strains have been isolated [33]. Emerging organisms like *Raoultella* species are likely to escape routine identification or be disregarded as insignificant contaminants despite their potential to cause infections which are complicated to manage due to MDR [34]. Limitations in diagnostic microbiological capacities might lead to delayed identification of emerging pathogens and extended AMR patterns, resulting in suboptimal patient care. In the light of increasing AMR among pathogens worldwide but also in resource-limited settings, strengthening of microbiological facilities and thus improved infection surveillance and control will lead to better hospital hygiene and infection control practices, which are needed for optimal outcome and containment of spreading resistance genes.

## Conclusions

This report shows evidence of an infection due to a MDR strain of *R. planticola* causing a SSI and intra-abdominal infection with the presence of the CTX-M-9 group ESBLs and is the first description of such an infection in Africa. Surgical treatment with intestinal leakage was the likely route of the infection caused by *R. planticola*. The source of the bacteria might be from gastrointestinal colonization as consequence of ineffective antibiotics utilization or from the hospital environmental. The absence of diagnostic facilities, limited awareness of treating physicians for the importance of microbiological culturing and AST, restricted availability of antibiotic substances for treatment of infections caused by MDR bacteria and lack of data for common local AMR patterns in resource limited settings is jeopardizing the success rate of empirical antibiotic treatment. These insufficiencies become more severe in patients with hospital-acquired infections and prolonged stay in a hospital. In general, the presented case serves to increase the awareness that rare bacterial species like *Raoultella* should be considered as a potential cause of nosocomial SSI with a high rate of AMR. This leads to the necessity of up-to-date guidelines for the management of intra-abdominal infections and for regular microbiological investigation of suitable clinical specimen in nosocomial infections.

In settings where colonization with MDR *Enterobacteriaceae* is common, surveillance and screening for colonization with MDR bacterial strains before invasive procedures and implementation or strengthening of antibiotic stewardship programs would contribute to successful patient care and should be considered.

## Data Availability

All data generated or analysed during this study are included in this published article [and its additional information files].

## Abbreviations

AMR: Antimicrobial resistance
AST: Antimicrobial susceptibility testing
ATRH: Asella Teaching and Referral Hospital
BP: Blood pressure
CS: Caesarean section
ESBL: Extended spectrum β-lactamase
MDR: Multi-drug resistant
PR: Pulse rate
RR: Respiratory rate
SSI: Surgical site infection
T: Body temperature
